# Ultrasonographic Evaluation of Cerebral Arterial and Venous Haemodynamics in Multiple Sclerosis: A Case-Control Study

**DOI:** 10.1371/journal.pone.0111486

**Published:** 2014-10-31

**Authors:** Pasquale Marchione, Manuela Morreale, Patrizia Giacomini, Chiara Izzo, Simona Pontecorvo, Marta Altieri, Silvia Bernardi, Marco Frontoni, Ada Francia

**Affiliations:** 1 Neurovascular Diagnosis Unit, Department of Medical and Surgical Sciences and Biotechnology – Section of Neurology, Sapienza, University of Rome, Rome, Italy; 2 Department of Clinical Neurosciences, Neurological Centre of Latium – Institute of Neurosciences, Rome, Italy; 3 Multiple Sclerosis Center, Department of Neurology and Psychiatry, Sapienza, University of Rome, Rome, Italy; 4 Istituto di Ricerca e Cura a Carattere Scientifico – Neuromed, Pozzilli (Isernia), Italy; University of California San Diego, United States of America

## Abstract

**Objective:**

Although recent studies excluded an association between Chronic Cerebrospinal Venous Insufficiency and Multiple Sclerosis (MS), controversial results account for some cerebrovascular haemodynamic impairment suggesting a dysfunction of cerebral autoregulation mechanisms. The aim of this cross-sectional, case-control study is to evaluate cerebral arterial inflow and venous outflow by means of a non-invasive ultrasound procedure in Relapsing Remitting (RR), Primary Progressive (PP) Multiple Sclerosis and age and sex-matched controls subjects.

**Material and Methods:**

All subjects underwent a complete extra-intracranial arterial and venous ultrasound assessment with a color-coded duplex sonography scanner and a transcranial doppler equipment, in both supine and sitting position by means of a tilting chair. Basal arterial and venous morphology and flow velocities, postural changes in mean flow velocities (MFV) of middle cerebral arteries (MCA), differences between cerebral venous outflow (CVF) in clinostatism and in the seated position (ΔCVF) and non-invasive cerebral perfusion pressure (CPP) were evaluated.

**Results:**

85 RR-MS, 83 PP-MS and 82 healthy controls were included. ΔCVF was negative in 45/85 (52.9%) RR-MS, 63/83 (75.9%) PP-MS (p = 0.01) and 11/82 (13.4%) controls (p<0.001), while MFVs on both MCAs in sitting position were significantly reduced in RR-MS and PP-MS patients than in control, particularly in EDSS≥5 subgroup (respectively, 42/50, 84% vs. 66/131, 50.3%, p<0.01 and 48.3±2 cm/s vs. 54.6±3 cm/s, p = 0.01). No significant differences in CPP were observed within and between groups.

**Conclusions:**

The quantitative evaluation of cerebral blood flow (CBF) and CVF and their postural dependency may be related to a dysfunction of autonomic nervous system that seems to characterize more disabled MS patients. It's not clear whether the altered postural control of arterial inflow and venous outflow is a specific MS condition or simply an “epiphenomenon” of neurodegenerative events.

## Introduction

Since first hysto-pathological descriptions of perivenous inflammation in Multiple Sclerosis (MS), vascular venous hypothesis has had mixed fortunes during the past century until more recent Zamboni's renaissance [1]–[6]. Although the potential therapeutic implications and the subsequent scientific and public controversies, two recent case-control studies on large cohort of patients failed to show an association between Chronic Cerebrospinal Venous Insufficiency (CCSVI) and MS [7]–[9]. Further evidences of non-disease-specific changes of cerebral venous flow (CVF) suggested a possible secondary nature of the venous drainage alterations in MS [10,11]. Nevertheless, the presence of some cerebrovascular haemodynamic impairments has been described, independently from CCSVI reports. Particularly, a widespread hypoperfusion in focal lesions and in both the grey and white matter in all clinical phenotypes of MS has been observed with single photon emission tomography (PET) and perfusion magnetic resonance imaging (pMRI) [12]–[15]. Moreover, contrast enhanced ultrasonography of the Internal Jugular Vein (IJV) with time-intensity curve analysis revealed alterations of CVF in MS [16]. Postural dependency of the CVF has been demonstrated in healthy subjects by Color-Doppler Sonography and seems to be reduced in MS patients [17]. Interestingly, arterial brain flow velocities (BFVs) also decreased more in MS patients than in controls during head up tilt, expecially in more disabled patients [18]. All these evidences suggested such an autonomic nervous system dysfunction that impaired cerebral autoregulation mechanisms during MS but the possible correlation between perfusion impairment and outflow alterations has not yet been investigated. The aim of this case-control and cross-sectional study is to evaluate cerebral arterial inflow and venous outflow by means of a wide non-invasive ultrasound procedure in both Relapsing-Remitting (RR-MS) and Primary Progressive (PP-MS) patients, representing two clinical opposites of the same disease.

## Materials and Methods

All subjects voluntarily participated the study after written informed consent concerning treatment of personal data, background and objective of the study, methodologies and duration of ultrasound assessment. Scientific/Ethic committee of Sapienza – University of Rome, Department of Neurology and Psychiatry approved this no-profit study.

### Study design

We designed a single-center, cross-sectional, blinded, case-control study including patients with defined diagnosis of RR-MS and PP-MS according to revised McDonald criteria [19] from Multiple Sclerosis Centre of the Department of Neurology and Psychiatry (Sapienza, University of Rome) and age and sex-matched genetically unrelated controls subjects from a General Practitioner database in the catchment area of the same Centre (HC). The study aimed to enroll RR-MS, PP-MS and healthy controls in a 1∶1∶1 ratio. Exclusion criteria were presence of relapse and steroid treatment in the 30 days before examination, secondary progressive form of MS (SP-MS), atherosclerotic disease, other neurological disorders (e.g., cerebrovascular disease, head trauma, vasculitis, transient global amnesia, history of alcohol and illicit drug abuse), history of cerebral congenital vascular malformations and pregnancy. To ensure blinded design, all the participants were instructed not to reveal their status during the examination and ultrasonographers approached the subjects already lying on the tilting chair in a quiet and dark room in order to avoid recognition of neurological signs. Medical personnel of Multiple Sclerosis Center who provide all the information to both patients and controls before ultrasound study performed clinical evaluation. At time of selection, standard demographic and clinical information were acquired by means of a structured questionnaire and clinical examination, including all data in a prearranged database which take into account age, sex, disease duration, clinical manifestations at onset and follow up, expanded disability status scale (EDSS) [20], detailed familial and personal medical history, ambulatory cardiovascular parameters, last laboratory examinations and current and previous therapy information. According to EDSS score, MS patients were further subdivided in two subgroups with an EDSS respectively <5 and ≥5. Before informed consent in line with the principles of the Declaration of Helsinki, all the participants were learned about background and objective of the study, methodologies and duration of ultrasound assessment. Clinical and ultrasonographic data were independently collected at the end of the study by different researchers who designed the study.

### Ultrasound protocol

All subjects underwent a complete extra and intracranial arterial and venous ultrasound assessment with a high-resolution color-coded duplex sonography scanner by means of high frequency (5–10 MHz) linear probe for the cervical veins (extracranial color-coded doppler sonography, ECDS) and a low frequency (1–3 MHz) phased-array probe for the intracranial veins (transcranial color-coded doppler sonography, TCDS) (LogiQ-Pro, General Electrics). A blinded transcranial assessment (transcranial doppler, TCD) were further performed with both a single 2–4 MHz probe for basal evaluation of Willis arteries and a double 2–4 MHz probe fixed with elastic head band for bilateral insonation of middle cerebral arteries (MCA) (MultiDopX, Compumedics Inc.). The examination was always performed with the subjects lying first in a supine position and then in a sitting position by means of a tilting chair with the head placed in a straight attitude in order to avoid flow alterations caused by unilateral or bilateral venous outflow obstruction. The probe was gently applied over the neck not to compress the cervical veins and to obtain reliable velocity values or cross-sectional area (CSA) measurements. Mean blood pressure and heart rate were continuously measured independently from position by means of a blood pressure monitor (2300 Finapress Ohmeda Medical)

### Extracranial assessment

A complete examination of common carotid (CCAs), internal carotid (ICAs) and vertebral (VAs) arteries was performed by means of both longitudinal and transverse B-mode insonation planes in clinostatism. CSAs of both CCAs and VAs have been estimated from the diameter of the vessels in transverse plane, assuming a circular vessel shape. Arterial global cerebral blood flow (CBF) was calculated offline as the sum of the products of arterial CSAs and the time averaged flow velocities over at least four heart cycles in both ICAs and VAs. The internal jugular veins (IJVs) and the vertebral veins (VVs) were bilaterally examined by means of longitudinal and transverse B-mode insonation planes in both clinostatism and orthostatism. Since the VVs commonly form a single vein only in the more basal cervical segments of C5–C6, venous CSAs have been estimated immediately over the upper bulb after assuming a circular vessel shape. A significant asymmetry of the venous CSA (>50%) in supine position was defined as anatomical variant. Reverted postural control of the main cerebral outflow pathway in the IJVs was obtained by subtracting the CSA measured in the supine from that in the sitting position (ΔCSA). The occurrence of a negative ΔCSA value (greater value in the sitting position) was defined as a possible marker of loss of postural control. Global CVF was the sum of the offline calculated products obtained by multiplying the venous CSA areas of both IJV and VV with the time averaged blood flow velocities assessed during short apnoea after a normal exhalation over at least 5 s in supine and sitting position [21,22]. The difference of CVF in supine and in seated position (ΔCVF) was calculated as a functional index of abnormal postural control of the venous outflow.

### Intracranial examination

First, the different segments of the arterial circle of Willis (M1–M2 segment of the middle cerebral artery [MCA], A1 segment of the anterior cerebral artery [ACA] and P1 and P2 segments of the posterior cerebral artery [PCA]) were insonated on both sides by means of TCD, recording only angle-corrected peak systolic flow velocities (PSVs), end diastolic flow velocities (EDVs) and mean flow velocities (MFVs). Velocimetric parameters on MCAs were further evaluated 90 s and 2 min after reaching the sitting position by means of double probe [23]. Breath-hold with short apnoea was used to test cerebrovascular reactivity (Breath Holding Index, BHI) by monitoring end-tidal expiratory CO2 as previously described (Datex Normocap 200). Briefly, the patients were instructed to hold their breath without Valsalva' maneuver for 14–30 seconds after an inspiration. The BHI was obtained by dividing the percent increase in MFV occurring during breath holding by the length of time (in seconds) the subjects hold their breath after a normal inspiration ([MFV at the end of breath holding – rest MFV]/rest MFV×100/s of breath holding) [24]. Cerebral perfusion pressure (CPP) in both supine and 90 s-sitting position was non-invasively assessed by means of TCD blood flow velocity waveform according to the subsequent formula: CPP = mean ABP×EDV/MFV+14 mmHg [25]. Then, intracranial arterial and venous system was examined both in supine and in sitting position through the transtemporal bone window by means of TCDS, after adjusting the system settings for the analysis of low-velocity signals during vein insonation (switched off filters, pulse repetition frequency-PRF ranging between 0.5 and 1.4 values, depth of insonation from 7 to 10 cm, accustomed color gain to the optimal signal-to-noise ratio). The basal vein of Rosenthal (BV) slightly upward the P2 segments of the PCA and the vein of Galen (VG) in the midline posterior to the pineal region on the ventricular plane were detected. In the same plane, the transverse sinuses (TS) is more laterally and deeply insonated, if possible, nearly the contralateral hyperechogenic skull with a flow direction away from the probe. The deep middle cerebral vein (dMCV) was located slightly posteriorly to the MCA adjacent to the branching into the M2 segments. The straight sinus (SRS) was measured in the middle third of the distance between pineal gland and internal occipital protuberance in the plane of the apex of the cerebellar tentorium and the internal occipital protuberance by upward rotation of the anterior tip of the probe. PSVs and EDVs of all paired venous vessels were measured bilaterally, not at junctions with other vessels to avoid changes of venous flow velocities at those points.

### Statistical analysis

Statistical analyses were performed using Statistical Package for Social Sciences (SPSS, version 15.1). Clinical and demographic data are expressed as the mean ± standard deviation (SD). Descriptive statistics and estimates of prevalence were performed by means of t tests. Differences among groups were tested for significance with the analysis of variance (ANOVA) and with the 2-sided Fisher's exact test. Values of p<0.05 were considered to be significant.

## Results

### Clinical and demographic data

Of the 180 enrolled patients, 173 subjects were eligible for statistical analysis (89 RR-MS and 84 PP-MS) of which 4 RR patients were excluded because of carotid atherosclerosis (1 man) and unavailable transtemporal bone windows (1 man and 2 women), while 1 PP woman abandoned the study because of a previously undiagnosed thyroid enlargement. 82 age and sex matched HCs were selected from general population according to exclusion criteria. Demographic and clinical characteristics of the enrolled groups are showed in table 1. No age and sex differences were found in any of the control or disease groups. Mean basal arterial pressure and beat-rate (in clinostatism) were similar in both groups. 64 of the 85 RR-MS and 18 of the 83 PP-MS patients were on disease-modifying therapy.

**Table 1:** Clinical and demographic data.

	RR n. 85	PP n. 84	HC n. 82	p
mean age ± SD (years)	39±13	42±14	41±12	n. s.
F∶M ratio	4∶1	3∶2	4∶1	n. s.
disease duration (years)	8±7	11±6	-	n. s.
EDSS	2.9±0.8	4.9±1.5	-	.01
basal MAP (mmHg)	96.7±3.9	95.1±4.1	97.3±3.6	n. s.
basal HR (beat/min.)	68±1.2	67±1.6	68±1.4	n. s.

### Arterial and Venous blood volume flow

**Figure 1:**
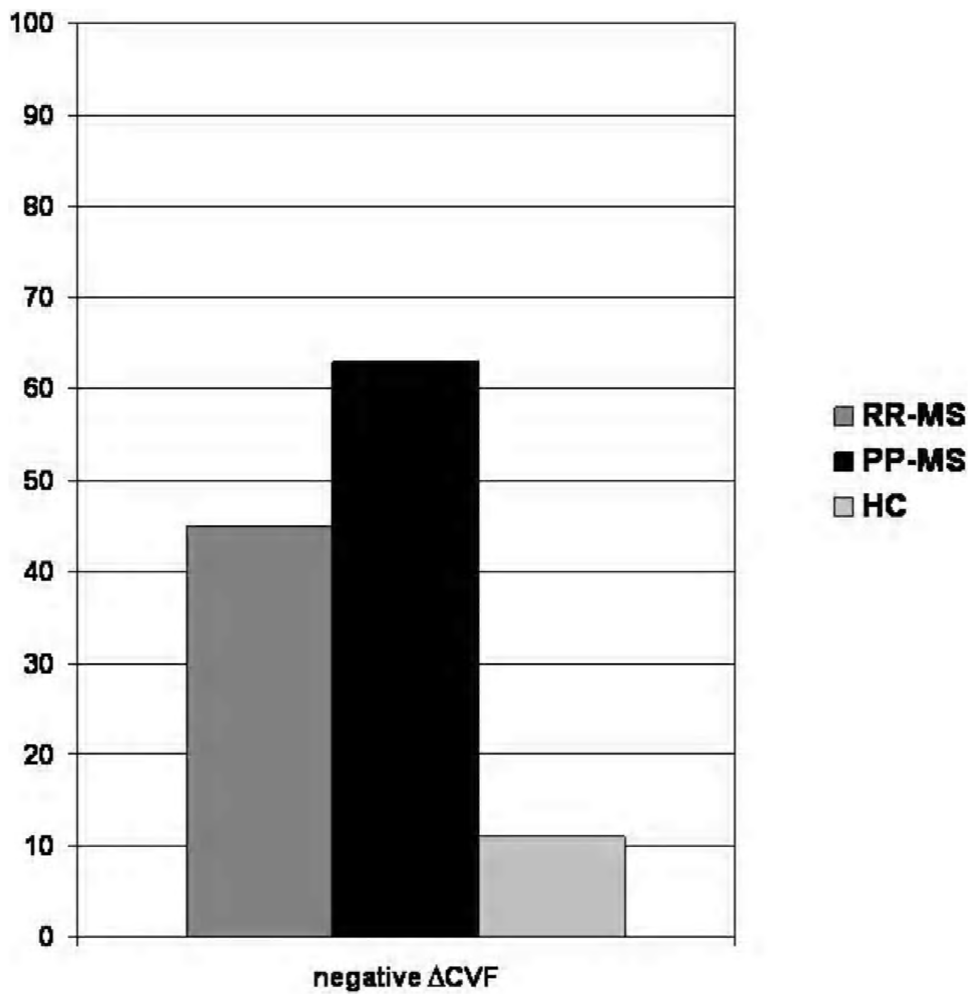
Negative ΔCVF distribution between groups.

A significant asymmetry of the venous CSA (>50%) in supine position were observed in 19 (22.3%) RR-MS, 17 (20.2%) PP-MS patients and 16 (19.5%) HCs with prevalent reduction of left IJV diameter. A negative ΔCSA value was observed in 3 (3.5%) RR-MS, 1 PP-MS (1.2%) and 3 (3.6%) controls. Mean global CBF and CVF values in clinostatism are showed in table 2. The slight reduction of both global CBF and mean total CVF resulted no statistically significant. ΔCVF was negative in 108/173 MS patients (62.4%) and 11/82 (13.4%) HCs (p<0.001). According to clinical phenotype, ΔCVF was negative in 45/85 (52.9%) RR-MS and 63/83 (75.9%) PP-MS (p = 0.01) (figure 1). Age and sex-related differences of CBF, CFV and ΔCVF were not statistically significant. There were no significant correlation among negative ΔCVF and significant asymmetry of venous CSA or negative ΔCSA value. A significantly higher prevalence of negative ΔCVF was observed in EDSS>5 subgroup (42/50, 84%) than in EDSS<5 subgroup (66/118, 55.9%) (p<0.01).

**Table 2:** Cerebral arterial and venous blood volume flow parameters.

	RR (ml/min)	PP (ml/min)	controls (ml/min)	P
global CBF	747±136	740±133	759±139	n. s.
mean total CVF	657±238	654±253	668±246	n. s.
CVF in IJVs	614±251	619±231	625±244	n. s.
CVF in VVs	39±32	37±30	44±34	n. s.

### Intracranial haemodynamic parameters

Mean values of PSVs, EDVs and MFVs on TCD examination were similar in all examined arteries between groups (table 3). Postural variations of MFVs in both MCAs were reported in table 4. In sitting position, mean values of MFVs on both MCAs were significantly reduced in RR-MS and PP-MS patients than in control either after 90 s or after 2 min. No significant differences were observed according to age, sex and disease duration. Mean MFV values after 90 s of sitting position were prevalently reduced in patients with EDSS ≥5 than in patients with EDSS<5 (48.3±2 cm/s vs. 54.6±3 cm/s, p = 0.01). BHI values were similar among three groups (RR-MS: 0.88±0.2; PP-MS = 0.86±0.4; HC: 0.88±0.3; p = 0.892). Postural variation of MAP and CPP was reported in table 5. No significant differences in MAP and non-invasive CPP were observed within and between group. Single cerebral veins were inconstantly detected by means of TCDS among subjects without differences between patients and controls and the relative percentages are reported in table 6. Significant reflux was not found in patients or controls and velocity values were within a normal range in all study groups.

**Table 3:** Basal arterial haemodynamic parameters.

	PSV (cm/s)		EDV (cm/s)		MFV (cm/s)		
	MS	HC	MS	HC	MS	HC	p
MCA-M1	75±14	76±11	46±10	47±9	62±12	63±10	n. s.
ACA-A1	63±7	64±8	33±5	30±6	48±6	47±7	n. s.
PCA-P1	57±6	60±7	19±4	18±5	38±5	39±6	n. s.

**Table 4:** Postural adaptation of MFVs on MCAs.

	RR-MS	PP-MS	HC	p
Basal (cm/s)	62.4±11	61.8±12	62.9±10	n. s.
90 s (cm/s)	52.2±6	51.7±5	60.4±7	0.01
2 min (cm/s)	55.1±7	56.3±6	61.5±11	0.01

**Table 5:** MAP and CPP parameters in supine and sitting position.

		MS	HC	P
MAP (mmHg)	supine	93.8±10	95.3±11	n. s.
	sitting	90.1±8	90.4±9	n. s.
CPP (mmHg)	supine	83±6	84.8±5	n. s.
	sitting	81.6±7	81.1±6	n. s.

**Table 6:** Percentage of cerebral vein detection by TCDS.

	MS	HC	P
BV	86%	87%	n. s.
VG	76%	74%	n. s.
dMCV	69%	70%	n. s.
TS	39%	38%	n. s.
SRS	45%	45%	n. s.

## Discussion

The idea of venous congestion as a possible contributor to pathogenesis of MS has been discussed for the past 40 years, but remained widely unappreciated by the scientific community [3]–[4], [6,26]. Until recently, no scientific attempt was undertaken to prove or disprove the hypothesis despite its potential therapeutic implications. Therefore, the exceptionally significant results presented by Zamboni et al generated extraordinary interest and controversy in both scientific and social communities [27]. According to the Author, CCSVI may play a significant role in MS etiology by means of a venous congestion and an impairment of iron accumulation [28]. Several repetition studies did not reach the same strict evidences and the reliability of the CCSVI-MS association has already been debated [5]–[7]. Moreover, proposed parameters from Zamboni et al has been considered questionable by some Authors [5,29]. “A swan song for CCSVI” has been sung by two recent large and unbiased studies that suggests no association of CCSVI to MS [7]–[9]. Nevertheless, some haemodynamic alterations of venous system in MS patients may represent a functional epiphenomenon of intracranial microvascular impairment. Despite a slight difference in global CBF e CFV between MS patients and HCs, basal arterial and venous haemodynamic features did not differ among groups. The results of this study suggested such an impairment of postural control of both arterial inflow and venous outflow in MS patients without affecting CCP and CVR to apnea. As suggested by Gonul et al, arterial response to postural variation is a reliable method to assess cerebral autoregulation and may depend on autonomic and neurocardiogenic control systems of cerebral perfusion [18]. Several studies observed an orthostatic intolerance in MS patients but the pathophysiological mechanisms has not been elucidated and the relationship with disease severity and progression is still an object of debate [30]–[34]. Since significant changes of non invasively measured MAP and HR in sitting position has not been observed in our series, an impairment of cerebral microvasculature may be suggested but the preserved endothelial-dependent cerebral autoregulation did not support this hypothesis. Moreover, CPP remains stable after 90 s of sitting position and no differences has been found between MS patients and HCs. In addition, postural adaptation of MFVs was not different among sex, age or disease duration groups while a significant association has been observed with clinical disability. More sympathetic rather than parasympathetic dysfunction appears early in the course of MS and correlates with disease progression and MS clinical disability [35], [36]. A noradrenergic dysfunction may determine an altered vasoconstriction that would explain the reduced postural control of arterial inflow independently from changes in arterial blood pressure (ABP) [37]. The same sympathetic impairment could increase venomotor tone with resulting abnormal compliance of venous vessel wall [30]. Although we did not observed significant differences in global CVF between patients and controls, an altered postural control of venous outflow was more prevalent in MS patients, particularly in PP phenotype and in more disabled patients. According to Monti et al, such an explanation may be a no efficient Spinal Epidural Veins outflow as an additional drainage pathway in the seated position [38]. However, the cerebral venous system has more anatomical variability than the intracranial system and is often asymmetrical. The IJVs are the prevalent extracranial outflow pathways in the supine position while redirection of venous blood flow towards the vertebral veins occurs in the upright position [17,22]. In this study, a significant prevalence of right IJV in supine position and no alteration in available intracranial veins have been observed in line with previous findings [22,39,40]. Nevertheless, we did not find any association among anatomical variability, positive ΔCSA and loss of postural venous drainage control. Therefore, any pathology (stenosis) affecting cranio-cervical or azygous venous system such as a IJV's valve incompetence seems not sustainable. Some methodological issues need to be stressed at this point. First, the patients reached head upright in sitting position on a tilting chair and not on a tilt bed assuming a constant MCA diameter. Second, the angle of insonation may change during head up tilt and could adversely affect the results. Furthermore, this is a single cross-sectional centre study with a single trained sonographer. Although patients with recent relapse or treated with corticosteroids have been excluded, the relative impact of disease progression and disease-modifying treatments are not exhaustively elucidated. As a matter of fact, despite several attempts of standardization, ultrasonographic techniques are operator-dependent and this is particularly true for venous assessment because of a wide anatomical and physiological variability of venous system and a high bias due to manual pressure. In addition, the interpretation of extracranial and intracranial venous studies is hampered by the fact that the interobserver and intra-observer reliability of the method is still variable [41]. On the contrary, the strength of this study is a meticulous blinded design and a relatively good sample size with a high percentage of PP-MS patients and a large amount of measured haemodynamic parameters. Considering RR-MS and PP-MS as two clinical opposites of the same disease, we excluded SP-MS patients in order to avoid possible confounding factors.

In conclusion, our study suggested that the quantitative evaluation of CBF and CVF and their postural dependency may be related to a dysfunction of autonomic nervous system that seems to characterize more disabled MS patients. It's not clear whether the altered postural control of arterial inflow and venous outflow is a specific MS condition or simply represents an “epiphenomenon” of neurodegenerative events. Further studies correlating ultrasonographic hemodynamic data with MR imaging lesion load and with perfusional parameters are needed in order to confirm our hypothesis.

## References

[1] Charcot JM (1868) Histology of “sclerose en plaque” [in French]. Gaz Hop Civils Milit (Paris) 41: 554–566.

[2] Putnam TJ (1933) The pathogenesis of multiple sclerosis: a possible vascular factor. N Engl J Med 209: 786–790.

[3] Schelling F (1986) Damaging venous reflux into the skull or spine: relevance to multiple sclerosis. Med Hypotheses 21: 141e8. 3641027 10.1016/0306-9877(86)90003-4

[4] Talbert DG (2008) Raised venous pressure as a factor in multiple sclerosis. Med Hypotheses 70: 1112e17. 18079069 10.1016/j.mehy.2007.10.009

[5] Zamboni P (2006) The big idea: iron-dependent inflammation in venous disease and proposed parallels in multiple sclerosis. J R Soc Med 99: 589–593. 17082306 10.1258/jrsm.99.11.589 PMC1633548

[6] D'haeseleer M, Cambron M, Vanopdenbosch L, De Keyser J (2011) Vascular aspects of multiple sclerosis. Lancet Neurol 10: 657–66. 21683931 10.1016/S1474-4422(11)70105-3

[7] Reekers J (2014) A Swan Song for CCSVI. Cardiovasc Intervent Radiol 37: 287–288. 24402646 10.1007/s00270-013-0833-6

[8] Comi G, Battaglia MA, Bertolotto A, Del Sette M, Ghezzi A, et al (2013) Italian multicentre observational study of the prevalence of CCSVI in multiple sclerosis (CoSMo study): rationale, design, and methodology. Neurol Sci 34: 1297–1307. 23344741 10.1007/s10072-012-1269-5 PMC3747324

[9] Traboulsee AL, Knox KB, Machan L, Zhao Y, Yee I, et al (2014) Prevalence of extracranial venous narrowing on catheter venography in people with multiple sclerosis, their siblings, and unrelated healthy controls: a blinded, case-control study. Lancet 383: 138–45. 24119384 10.1016/S0140-6736(13)61747-X

[10] Ertl-Wagner B, Koerte I, Kümpfel T, Blaschek A, Laubender RP, et al (2012) Non-specific alterations of cranio-cervical venous drainage in multiple sclerosis revealed by cardiac-gated phase-contrast MRI. Mult Scler 18: 1000–7. 22194216 10.1177/1352458511432742

[11] Garaci FG, Marziali S, Meschini A, Fornari M, Rossi S, et al (2012) Brain hemodynamic changes associated with chronic cerebrospinal venous insufficiency are not specific to multiple sclerosis and do not increase its severity. Radiology 265: 233–9. 22915599 10.1148/radiol.12112245

[12] Sun X, Tanaka M, Kondo S, Okamoto K, Hirai S (1998) Clinical significance of reduced cerebral metabolism in multiple sclerosis: a combined PET and MRI study. Ann Nucl Med 12: 89–94. 9637279 10.1007/BF03164835

[13] Law M, Saindane AM, Ge Y, Babb JS, Johnson G, et al (2004) Microvascular abnormality in relapsing remitting multiple sclerosis: perfusion MR imaging findings in normal-appearing white matter. Radiology 231: 645–652. 15163806 10.1148/radiol.2313030996

[14] Inglese M, Park SJ, Johnson G, Babb JS, Miles L, et al (2007) Deep gray matter perfusion in multiple sclerosis: dynamic susceptibility contrast perfusion magnetic resonance imaging at 3 T. Arch Neurol 64: 196–202. 17296835 10.1001/archneur.64.2.196

[15] Varga AW, Johnson G, Babb JS, Herbert J, Grossman RI, et al (2009) White matter hemodynamic abnormalities precede sub-cortical gray matter changes in multiple sclerosis. J Neurol Sci 282: 28–33. 19181347 10.1016/j.jns.2008.12.036 PMC2737614

[16] Mancini M, Lanzillo R, Liuzzi R, Di Donato O, Ragucci M, et al (2014) Internal Jugular Vein Blood Flow in Multiple Sclerosis Patients and Matched Controls. PLoS ONE 9: e92730. 24675965 10.1371/journal.pone.0092730 PMC3968019

[17] Valdueza JM, von Munster T, Hoffman O, Schreiber SJ, Einhaupl KM (2000) Postural dependency of the cerebral venous outflow. Lancet 355: 200–201. 10.1016/s0140-6736(99)04804-7 10675123

[18] Gonul M, Asilb T, Balcib K, Celikb Y, Turgutb N, et al (2008) Changing cerebral blood flow velocity detected by transcranial Doppler ultrasound during head up tilt in patients with multiple sclerosis. European Journal of Neurology 15: 725–729. 18505409 10.1111/j.1468-1331.2008.02179.x

[19] Polman CH, Reingold SC, Banwell B, Clanet M, Cohen JA, et al (2011) Diagnostic criteria for multiple sclerosis: 2010 Revisions to the McDonald criteria. Ann Neurol 69: 292–302. 21387374 10.1002/ana.22366 PMC3084507

[20] Kurtzke JF (1983) Rating neurologic impairment in multiple sclerosis: an expanded disability status scale (EDSS). Neurology 33: 1444–1452. 6685237 10.1212/wnl.33.11.1444

[21] Dörfler P, Puls I, Schliesser M, Mäurer M, Becker G (2000) Measurement of cerebral blood flow volume in extra cranial sonography. J Cereb Blood Flow Metab 20: 269–271. 10698063 10.1097/00004647-200002000-00007

[22] Doepp F, Schreiber SJ, von Münster T, Rademacher J, Klingebiel R, et al (2004) How does the blood leave the brain? A systematic ultrasound analysis of cerebral venous drainage patterns. Neuroradiology 46: 565–570. 15258709 10.1007/s00234-004-1213-3

[23] Heckmann JG, Hilz MJ, Hagler H, Muck-Weymann M, Neundorfer B (1999) Transcranial Doppler sonography during acute 80 degrees head-down tilt (HDT) for the assessment of cerebral autoregulation in humans. Neurology Research 21: 457–462. 10439426

[24] Silvestrini M, Vernieri F, Pasqualetti P, Matteis M, Passarelli F, et al (2000) Impaired cerebral vasoreactivity and risk of stroke in patients with asymptomatic carotid artery stenosis. JAMA 283: 2122–2127. 10791504 10.1001/jama.283.16.2122

[25] Czosnyka M, Matta BF, Smielewski P, Kirkpatrick PJ, Pickard JD (1998) Cerebral perfusion pressure in head-injured patients: a non-invasive assessment using transcranial Doppler ultrasonography. J Neurosurg 88: 802–8. 9576246 10.3171/jns.1998.88.5.0802

[26] Allen IV (1981) The pathology of multiple sclerosis fact, fiction and hypothesis. Neuropathol Appl Neurobiol 7: 169. 7242846 10.1111/j.1365-2990.1981.tb00087.x

[27] Tan IL, van Schijndel RA, Pouwels PJ, van Walderveen MA, Reichenbach JR, et al (2000) MR venography of multiple sclerosis. AJNR Am J Neuroradiol 21: 1039–42. 10871010 PMC7973892

[28] Zamboni P, Galeotti R, Menegatti E, Malagoni AM, Tacconi G, et al (2009) Chronic cerebrospinal venous insufficiency in patients with multiple sclerosis. J Neurol Neurosurg Psychiatry 80: 392–399. 19060024 10.1136/jnnp.2008.157164 PMC2647682

[29] Baracchini C, Valdueza JM, Del Sette M, Baltgaile G, Bartels E, et al (2012) CCSVI and MS: a statement from the European Society of neurosonology and cerebral hemodynamics. J Neurol 259: 2585–9. 22648477 10.1007/s00415-012-6541-3

[30] Valdueza JM, Doepp F, Schreiber SJ, van Oosten BW, Schmierer K, et al (2013) What went wrong? The flawed concept of cerebrospinal venous insufficiency. J Cereb Blood Flow Metab 33: 657–68. 23443168 10.1038/jcbfm.2013.31 PMC3652697

[31] Flachenecker P, Wolf A, Krauser M, Hartung HP, Reiners K (1999) Cardiovascular autonomic dysfunction in multiple sclerosis: correlation with orthostatic intolerance. Journal of Neurology 246: 578–586. 10463360 10.1007/s004150050407

[32] Saari A, Tolonen U, Pääkkö E, Suominen K, Pyhtinen J, et al (2004) Cardiovascular autonomic dysfunction correlates with brain MRI load in MS. Clinical Neurophysiology 115: 1473–1478. 15134718 10.1016/j.clinph.2004.01.012

[33] Lachenecker P, Reiners K, Krauser M, Wolf A, Toyka KV (2001) Autonomic dysfunction in multiple sclerosis is related to disease activity and progression of disability. Multiple Sclerosis 7: 327–334. 11724449 10.1177/135245850100700509

[34] Vita G, Fazio MC, Milone S, Blandino A, Salvi L, et al (1993) Cardiovascular autonomic dysfuction in multiple sclerosis is likely related to brainstem lesions. Journal of Neurological Science 120: 82–86. 10.1016/0022-510x(93)90029-x 8289084

[35] Frontoni M, Fiorini M, Strano S, Cerutti S, Giubilei F, et al (1996) Power spectrum analysis contribution to the detection of cardiovascular dysautonomia in multiple sclerosis. Acta Neurologica Scandinavica 93: 241–245. 8739432 10.1111/j.1600-0404.1996.tb00514.x

[36] Flachenecker P, Reiners K, Krauser M, Wolf A, Toyka KV (2001) Autonomic dysfunction in multiple sclerosis is related to disease activity and progression of disability. Mult Scler 7: 327–34. 11724449 10.1177/135245850100700509

[37] Labuz-Roszak B, Pierzchala K (2007) Difficulties in the diagnosis of autonomic dysfunction in multiple sclerosis. Clin Auton Res 17: 375–7. 17955330 10.1007/s10286-007-0443-y

[38] Wilson TD, Shoemaker JK, Kozak R, Lee TY, Gelb AW (2005) Reflex-mediated reduction in human cerebral blood volume. J Cereb Blood Flow Metab 25: 136–43. 15678119 10.1038/sj.jcbfm.9600015

[39] Monti L, Menci E, Ulivelli M, Cerase A, Bartalini S, et al (2011) Quantitative Colour-Doppler-Sonography Evaluation of Cerebral Venous Outflow: A Comparative Study between Patients with Multiple Sclerosis and Controls. PLoS ONE 6: e25012. 21966398 10.1371/journal.pone.0025012 PMC3178581

[40] Schaller B (2004) Physiology of cerebral venous blood flow: from experimental data in animals to normal function in humans. Brain Res Brain Res Rev 46: 243–60. 15571768 10.1016/j.brainresrev.2004.04.005

[41] Stolz E, Babacan SS, Bödeker RH, Gerriets T, Kaps M (2001) Interobserver and intraobserver reliability of venous transcranial color-coded flow velocity measurements. J Neuroimaging 11 4: 385–92. 11677878 10.1111/j.1552-6569.2001.tb00067.x

